# Genetic diversity of Bovine Viral Diarrhea Virus from cattle in Chile between 2003 and 2007

**DOI:** 10.1186/s12917-018-1641-7

**Published:** 2018-10-19

**Authors:** Astrid Donoso, Felipe Inostroza, María Celedón, José Pizarro-Lucero

**Affiliations:** Laboratory of Animal Virology, Department of Animal Preventive Medicine, Faculty of Livestock and Veterinary Sciences, University of Chile, Av. Santa Rosa, 11735 Santiago, Chile

**Keywords:** *Bovine viral diarrhea virus*, *Pestivirus*, BVDV, Genetic diversity, 5’UTR, E2

## Abstract

**Background:**

*Bovine Viral Diarrhea Virus* causes significant economic losses in cattle. BVDV has high genomic diversity, with two species, BVDV-1 and BVDV-2, and at least twenty-one subgenotypes for BVDV-1 and four subgenotypes for BVDV-2. Vaccines are important tools to reduce the economic losses caused by this virus. However, vaccine strains must correspond to the antigenic profile of the viruses present in the region where the vaccine is applied. A restricted phylogenetic study with 14 viruses isolated from cattle between 1993 and 2001 showed that the genetic profile of BVDV in Chile consisted of viruses of both species and sub-genotypes 1a, 1b, 1c (currently 1j) and 2a. To determine more accurately the genetic profile of BVDV in Chile, in this study a larger number of viruses obtained from bovines between 2003 and 2007 were typed.

**Results:**

The study was performed using partial sequences from the 5′ noncoding region (5’UTR) and E2 coding region of the viral genome of thirty-five Chilean viruses isolated from geographic regions that have 84.6% of the Chilean cattle. All tested viruses belonged to species BVDV-1. Eighteen viruses belonged to BVDV-1j subgenotype (51.4%), twelve belonged to BVDV-1b (34.3%) and five belonged to BVDV-1a (14.3%). The Chilean BVDV-1j viruses showed low genetic diversity, both among themselves and with the BVDV-1j present in other regions of the world. This could be explained by a relatively recent introduction of this viral subgenotype in cattle, which agrees with its low geographical distribution worldwide. Otherwise, Chilean BVDV-1b viruses grouped into a single cluster, different even than the viruses present in Argentina and Brazil, countries geographically close to Chile, a process of local evolution that could generate antigenic differences between the Chilean viruses and the viruses used as vaccine strains.

**Conclusions:**

The high presence of viruses of the BVDV-1j subgenotype, which show major antigenic differences with BVDV-1a and BVDV-1b subgenotypes used in the commercial vaccines, suggest that BVDV-1j viruses could be an emergent subgenotype of BVDV in cattle in South America and suggest evaluating an update of the vaccines used in Chile.

## Background

The *Bovine Viral Diarrhea Virus* (BVDV) is a virus of worldwide distribution, which causes a wide variety of clinical symptoms in cattle, being recognized as the viral agent that causes the main economic losses in the global cattle industry [1, 2].

In immunocompetent animals, BVDV usually is associated with respiratory and gastrointestinal diseases of different severity, hemorrhagic syndrome, and reproductive problems, such as infertility. BVDV also causes immunosuppression, which increases the severity of diseases caused by other pathogens. The virus is able to cross the placenta infecting the fetus, causing embryonic reabsorption, fetal mummification, abortion and congenital malformations, especially of the central nervous system. Fetal infection with a non-cytopathic virus in the first trimester of pregnancy causes persistently infected animals with BVDV (PI), which are the main source of infection of susceptible animals in the herd. Superinfection of PI animals with a cytopathic BVD virus very close antigenically with the non-cytopathic BVD virus produces the fatal bovine viral diarrhea-mucosal disease (BVD-MD) [3–6].

*Bovine Viral Diarrhea Virus* (BVDV) is a member of the genus *Pestivirus*, family *Flaviviridae*, which includes four specie: *Bovine Viral Diarrhea Virus 1* (BVDV-1), *Bovine Viral Diarrhea Virus 2* (BVDV-2), *Classical Swine Fever Virus* and *Border Disease Virus* [7]. The genome of BVDV is a single-stranded RNA of positive polarity, approximately 12.3 Kb in length. The genome contains a single open reading frame that encodes 11–12 viral proteins, flanked by a 5 ‘and 3’ untranslated region [7]. Genetic typing has classified the BVDV isolates into two species, *Bovine Viral Diarrhea Virus 1* (BVDV-1) and *Bovine Viral Diarrhea Virus 2* (BVDV-2), with at least 21 subgenotypes of BVDV-1 (1a-1u) and four subgenotypes of BVDV-2 (2a-2d) [7–14]. BVDV-2 strains were initially isolated from acute hemorrhagic disease outbreaks in North America, but later it could be established that both viral species of BVDV cause similar clinical symptoms. BVDV-1 strains are distributed worldwide, while the BVDV-2 strains are present mainly in the United States, Canada and South America, with few reports of their presence in Europe and Japan [10, 15–20]. Recently, an atypical pestivirus (HoBi-like) isolated from cattle and water buffaloes has been proposed as a new species (BVDV-3) based on phylogenetic analyzes [8, 21].

BVDV infection is widespread among Chilean cattle, with a serological prevalence ranging from 59.7 to 69.2% in unvaccinated animals from different regions of the country [22, 23]. To design an effective vaccination strategy against the BVDV virus in Chile, the vaccine strains must match the genetic profile of the viruses that infect Chilean cattle for optimal protection [24, 25]. In 2006, a phylogenetic study with 14 viruses isolated from cattle between 1993 and 2001 showed that the genetic profile of BVDV in Chile consisted of viruses of both species and subgenotypes 1a, 1b, 1c (currently 1j) and 2a, with a high prevalence of sub-type 1b (42.9%) [20]. The objective of this study was to determine more precisely the genetic profile of BVDV in Chile, with a greater number of viruses obtained from bovines between 2003 and 2007.

## Methods

### Samples

The thirty-five viruses included in this study were obtained from sera of calves and adult cattle and tissue of bovine fetuses from different regions of central and southern Chile from 2003 to 2007. Twenty-five viruses were isolated from sera of asymptomatic calves and adult cattle or those suffering enteric, respiratory or reproductive problems in the Metropolitana, Maule, Bio-Bio, Araucanía, De Los Rios and De Los Lagos Regions, which have 84.6% of the Chilean cattle [26]. Ten viruses were isolated from organ suspensions of bovine fetuses in the Metropolitana and Araucanía Regions. The list of the BVDV viruses examined in this study is shown in Table 1.

**Table 1:** Chilean BVDV field isolates studied in this work

Isolate	Year	Sample	Region	Clinical symptoms	Accession number		Type
					5’UTR	E2	
CHL/914	2005	LN	Araucanía	Abortion	JF759955	JF776638	BVDV-1b
CHL/916	2003	Serum	de los Ríos	PI	JF759940	JF776639	BVDV-1a
CHL/917	2003	Serum	de los Lagos	PI	JF759946	JF776640	BVDV-1b
CHL/918	2003	Serum	de los Lagos	PI	JF759944	JF776641	BVDV-1b
CHL/919	2003	Serum	de los Lagos	PI	JF759945	JF776642	BVDV-1b
CHL/920	2003	Serum	de los Lagos	PI	JF759947	JF776643	BVDV-1b
CHL/921	2003	Serum	de los Lagos	PI	JF759941	JF776644	BVDV-1a
CHL/927	2006	Serum	Bío-Bío	RD	JF759926	MH078039	BVDV-1j
CHL/928	2006	Serum	Bío-Bío	RD	JF759932	MH078040	BVDV-1j
CHL/939	2006	Serum	Bío-Bío	RD	JF759931	–	BVDV-1j
CHL/958	2006	Serum	Bío-Bío	RD	JF759928	MH078041	BVDV-1j
CHL/971	2006	Serum	Bío-Bío	RD	JF759937	–	BVDV-1j
CHL/972	2006	Serum	Bío-Bío	RD	JF759930	MH078042	BVDV-1j
CHL/992	2006	Serum	Bío-Bío	RD	JF759956	MH078043	BVDV-1j
CHL/1014	2006	Serum	Bío-Bío	RD	JF759929	–	BVDV-1j
CHL/1025	2007	Serum	Metropolitana	RD	JF759948	JF776645	BVDV-1b
CHL/1061	2007	Serum	Metropolitana	RD	JF759949	–	BVDV-1b
CHL/1068	2007	Serum	Metropolitana	RD	JF759942	JF776646	BVDV-1a
CHL/1071	2003	Serum	Araucanía	Diarrhea	JF759950	–	BVDV-1b
CHL/1076	2007	Serum	Araucanía	None	JF759951	–	BVDV-1b
CHL/1078	2007	Serum	Araucanía	Abortion	JF759936	MH078044	BVDV-1j
CHL/1086	2007	Serum	Araucanía	None	JF759954	–	BVDV-1b
CHL/1087	2007	Serum	Maule	None	JF759935	MH078045	BVDV-1j
CHL/1091	2007	Serum	Bío-Bío	None	JF759922	JF776648	BVDV-1a
CHL/1092	2007	Serum	Maule	Abortion	JF759952	–	BVDV-1b
CHL/1109	2007	Serum	Bío-Bío	None	JF759953	–	BVDV-1b
CHL/P22	2006	Lung	Metropolitana	None	JF759923	–	BVDV-1j
CHL/P23	2006	Lung	Metropolitana	None	JF759927	–	BVDV-1j
CHL/P24	2006	Lung	Metropolitana	None	JF759934	–	BVDV-1j
CHL/P26	2006	Lung	Metropolitana	None	JF759938	–	BVDV-1j
CHL/P28	2006	Lung	Metropolitana	None	JF759943	–	BVDV-1a
CHL/P30	2006	Lung	Metropolitana	None	JF759939	MH078046	BVDV-1j
CHL/P31	2006	Lung	Metropolitana	None	JF759933	–	BVDV-1j
CHL/P32	2006	Lung	Metropolitana	None	JF759924	–	BVDV-1j
CHL/P33	2006	Lung	Metropolitana	None	JF759925	–	BVDV-1j

*LN* Lymph Node, *PI* Persistent Infection, *RD* Reproductive Disorder

The viruses were propagated on monolayers of MDBK cells line (ATCC CCL-22), determined to be free of BVDV and grown in Eagle’s Minimum Essential Medium supplemented with 10% equine serum. All infections were monitored by direct immunofluorescence (DIF) assay. DIF was performed in acetone-fixed cells, using a FITC-conjugated anti-BVDV polyclonal serum (Central Veterinary Laboratory, Addlestone, Surrey, UK). All viruses were of the non-cytopathic biotype.

### Primers

The 5’UTR region corresponding to positions 108 to 395 according to the NADL genome sequence was amplified using the panpestivirus-specific primers 324/326 [27]. This pair of primers is highly sensitive for BVDV-1 and BVDV-2 viruses, but it is less sensitive for HoBi-like viruses, which may limit the detection of Hobi-like viruses in this study [21].

To confirm the data in 5’UTR, a 700 bp DNA fragment corresponding to positions 2754 to 3453 of the E2 region, according to the NADL genome sequence, was amplified with the primers E2F (5’-ACTTTGAATTTGGACTYTGCC-3′, nucleotides 2754–2774 of the NADL genome) and E2R (5’-TCCAGGTCAAACCARTATTG-3′, nucleotides 3453–3434 of the NADL genome) for selected viruses.

### RT-PCR

Total RNA was obtained from infected cells using Trizol LS (Invitrogen) according to the manufacturer’s instructions. The RNA was suspended in 50 μl DEPC-treated water. Amplification of viral RNA by RT-PCR was carried out in a SuperScript III One-Step RT-PCR System with Platinum Taq DNA Polymerase (Invitrogen), according to the manufacturer’s instructions, under the following conditions: 1) 5’-UTR Region: 12.5 μl 2x Reaction Mix, 0.2 mM dNTP, 2 mM MgSO_4_, 10 pmol each primer 324 and 326, 5 μl heat-denatured RNA (2 min at 94 °C), and 1 μl SuperScript III RT / Platinum Taq Mix. Synthesis of cDNA was performed in 25 μl final volume for 30 min at 55 °C. After reverse transcription, the reaction was heated in a thermocycler for 3 min at 94 °C and then submitted to 30 cycles of amplification. The amplification conditions were 30 s at 94 °C, 1 min at 55 °C and 1 min at 68 °C. 2) E2 Region: 12.5 μl 2x Reaction Mix, 0.2 mM dNTP, 1.6 mM MgSO_4_, 10 pmol each primer E2F and E2R, 5 μl heat-denatured RNA (2 min at 94 °C), and 1 μl SuperScript III RT/Platinum Taq Mix. Synthesis of cDNA was performed in 25 μl final volume for 30 min at 50 °C. After reverse transcription, the reaction was heated in a thermocycler for 3 min at 94 °C and then submitted to 30 cycles of amplification. The amplification conditions were 30 s at 94 °C, 1 min at 50 °C and 1 min at 68 °C. Amplified products were visualized by electrophoresis in 2% agarose gel run in 90 mM Tris, 90 mM boric acid, 2 mM EDTA at 18 V/cm of gel and stained with ethidium bromide.

### Genes sequencing and genetic analysis

The amplified products were purified using the QIAquick PCR purification kit (Qiagen) and DNA sequencing was performed on an ABI-310 automated sequencer using dideoxy-sequencing chemistry utilizing the Big Dye Terminator Cycle Sequencing with Ampli Taq DNA polymerase (Applied Biosystems). Both strands of each PCR product were sequenced in triplicate from amplified products obtained from individual amplifications.

Phylogenetic reconstructions for genetic typing were compiled using a 230 nucleotide region of the 5’-UTR (nucleotides 142–371 of the NADL genome) and a 420 nucleotide region of E2 (nucleotides 2879–3298 of the NADL genome). Nucleotide sequences from representative isolates of previously identified phylogenetic groups of BVDV were included in the phylogenetic analysis.

The nucleotide sequences obtained from each region were aligned using the MUSCLE program [28], then a matrix of distances for Kimura’s two-parameter model was generated, and the phylogenetic analysis was performed using the maximum likelihood method from the MEGA version 7 program [29]. The robustness of the phylogenetic analysis and significance of the branching order were determined by a bootstrap analysis carried out on 1000 replicates, which showed only bootstrap values of ≥50%. Sequence Demarcation Tool (SDT) v1.2 software was used to calculate pairwise identities using MUSCLE alignments for E2 nucleotide sequences of some viruses [30], and shown in matrix format. To analyze the differences at the molecular level of the E2 glycoprotein of the Chilean BVDV isolates with viruses of BVDV-1a, BVDV-1b and BVDV-1j subgenotypes from Argentina, Brazil and other regions of the world, the deduced amino acid sequences were aligned using MUSCLE and the pattern of amino acid substitutions near Domain C (amino acids 843 to 870), an important antigenic site of BVDV, was analyzed.

## Results

**Fig. 1:**
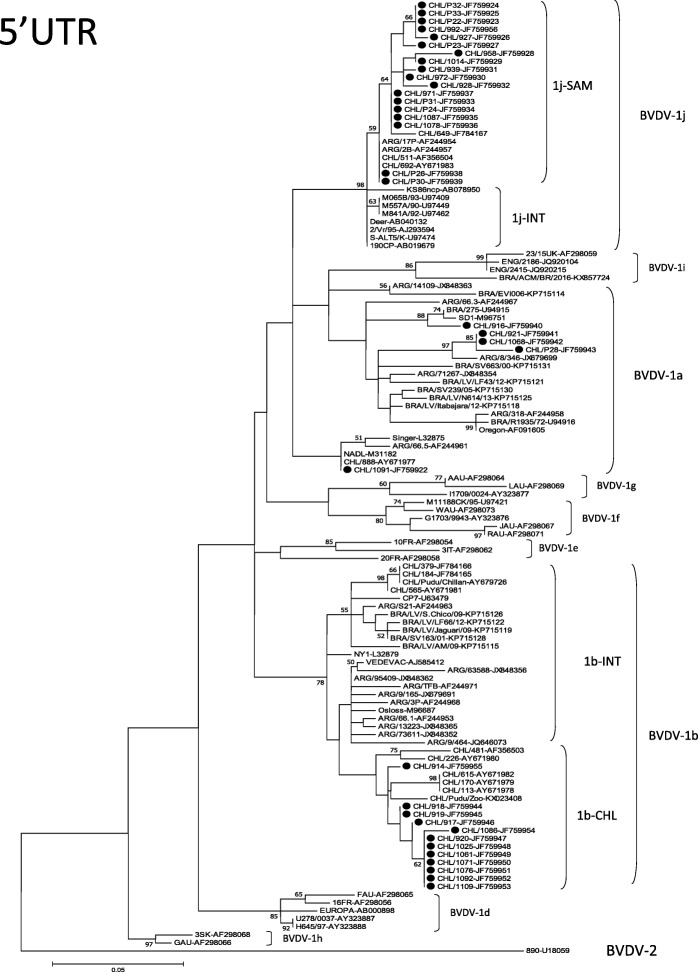
Phylogenetic analysis of Chilean BVDV isolates based on partial 5’-Untranslated Region (5’UTR). The phylogenetic tree were prepared using the Maximum Likelihood method and numbers over branches indicate the percentage of 1000 bootstrap replicates that support each phylogenetic branch. The GenBak accession numbers are indicated after the name of the BVDV strains. The Chilean viruses of this study are marked with circles

The 5’UTR phylogenetic tree was constructed using the nucleotide sequences of the 35 Chilean BVDV viruses of this study and BVDV reference strains from GenBank (Fig. 1). The accession numbers of the sequences obtained during this study are given in Table 1.

On the basis of phylogenetic analysis of 5’UTR, all 35 BVDV isolates belonged to species BVDV-1. The percentage identities (PID) among all the Chilean BVDV-1 viruses were 88–100%. Eighteen Chilean viruses grouped in the BVDV-1j subgenotype (51.4%), at a confidence level of 98% by bootstrap analysis. The PID among the Chilean BVDV-1j viruses were 97–100%. The BVDV-1j subgenotype was subdivided into two clusters. All the Chilean viruses grouped together with the Argentinean isolates 2B and 17P (cluster 1j-SAM). The other cluster was composed of BVDV-1j viruses isolated in Europe, Africa and Asia (cluster 1j-INT). Twelve Chilean viruses grouped in BVDV-1b subgenotype (34.3%), at a confidence level of 78% by bootstrap analysis. The PID among the Chilean BVDV-1b viruses were 96–100%. This subgenotype was subdivided into two clusters. All the Chilean viruses of this study were grouped with the viruses CHL/113, CHL/170, CHL/226, CHL/481 and CHL/615, also isolated from bovines in Chile [20], and the CHL/Pudu/Zoo isolated from pudú (*Pudu puda*), an indigenous deer of Chile from a Chilean Zoo, [31], forming a cluster only with Chilean viruses (cluster 1b-CHL). By contrast, the Chilean BVDV-1b viruses CHL/184, CHL/379, CHL/565 [20] and the CHL/Pudu-Chillan isolated from a free-ranging pudú [32] grouped in a different cluster with BVDV-1b viruses from other regions of the world (European, American, Argentinean, Brazilian) (cluster 1b-INT). Five Chilean viruses grouped in the BVDV-1a subgenotype (14.3%) with PID among them of 92–100%. The CHL/1091 virus showed a sequence identity of 100% with the NADL strain in the analyzed region of 5’UTR. There was no obvious association of any subgenotype with geographical location.

**Fig. 2:**
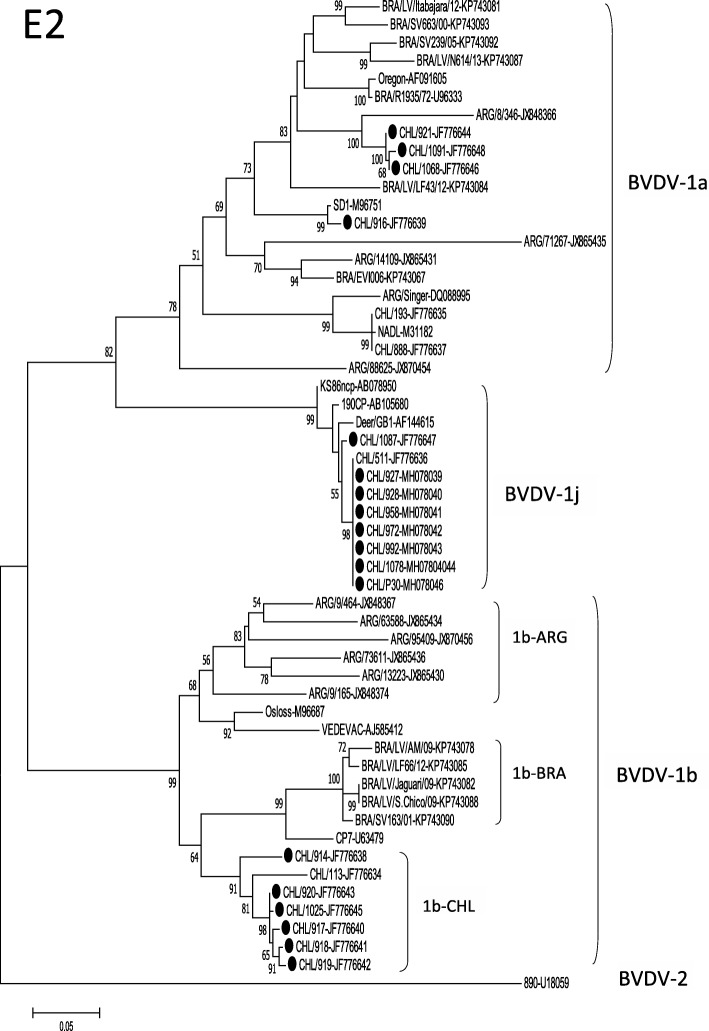
Phylogenetic analysis of Chilean BVDV isolates based on partial E2 coding region (E2). The phylogenetic tree was prepared using the Maximum Likelihood method and numbers over branches indicate the percentage of 1000 bootstrap replicates that support each phylogenetic branch. The GenBak accession numbers are after the name of the BVDV strains. The Chilean viruses of this study are marked with circles

To confirm the phylogenetic analysis obtained with the 5’UTR region, E2 genes of eighteen Chilean viruses of this study belonging to the different subgenotypes were sequenced, and the E2 phylogenetic tree was constructed using the nucleotide sequences of BVDV reference strains from GenBank (Fig. 2). The accession numbers of the sequences obtained are given in Table 1.

The results obtained with the E2 phylogenetic analysis showed the same classification as that observed using the 5’UTR nucleotide sequences, with the viruses conserving their type. The Chilean BVDV-1j, BVDV-1b, and BVDV-1a viruses grouped at a confidence level of 99%, 99%, and 78%, respectively, by bootstrap analysis. Chilean BVDV-1b viruses were grouped in a cluster only with Chilean viruses (cluster 1b-CHL) clearly apart from Brazilian and Argentinean viruses, with bootstrap values of 99% and 68%, respectively.

**Fig. 3:**
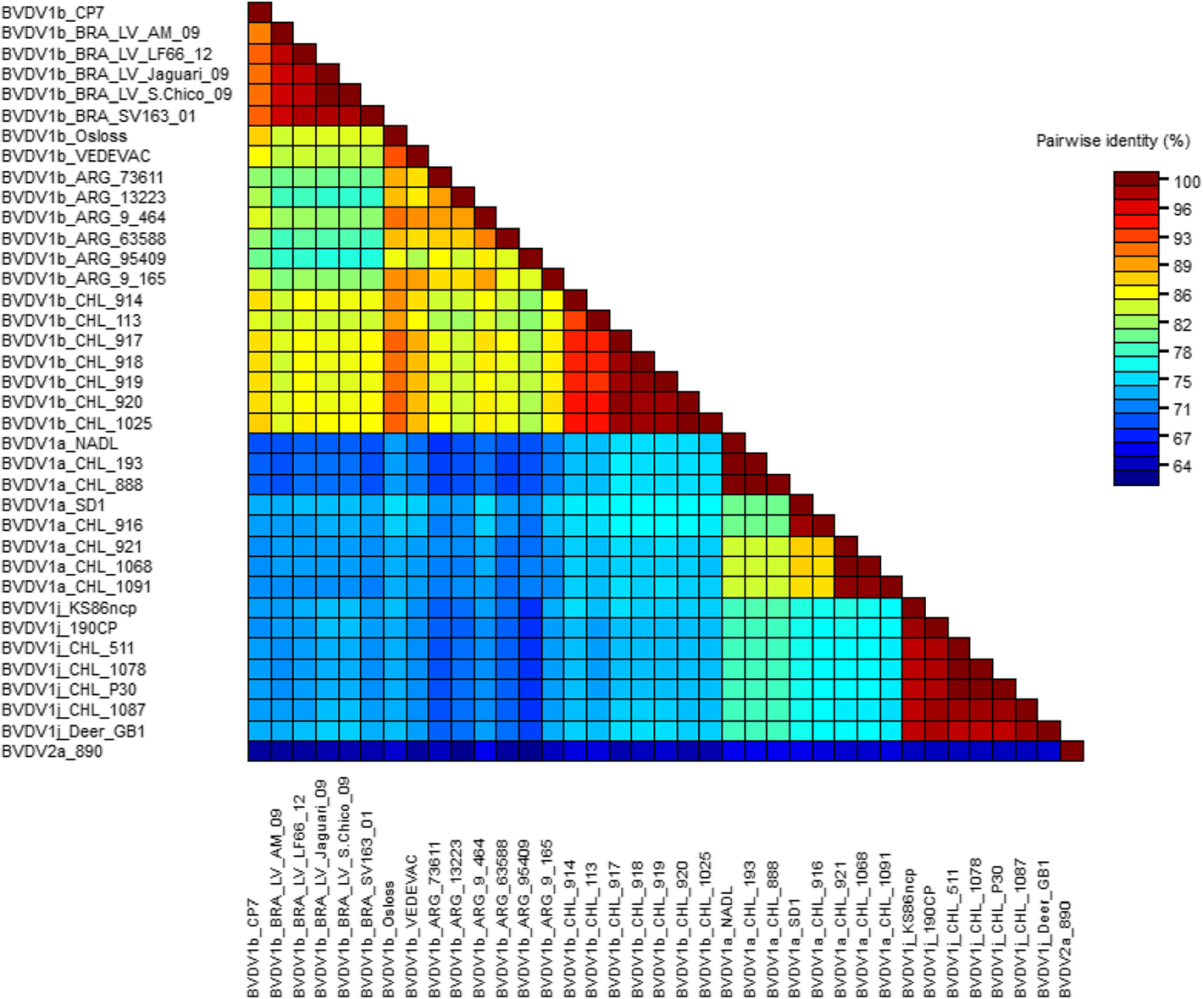
SDT matrix of pairwise identity scores generated by alignment of a 420 bp fragment of the E2 gene for 37 BVDV viruses. Each colored cell represents the percentage of identity between two nucleotide sequences, one horizontally and the other vertically, that intersect in the cell. A figure indicating the correspondence between pairwise identities and the colors is included

The pairwise identity matrix of selected E2 nucleotide sequences was calculated using SDT v1.2 software (Fig. 3). The matrix showed that the sequence identity among the Chilean BVDV-1a viruses isolated in this study ranged from 87 to 100%, between the Chilean BVDV-1b viruses ranged from 93 to 100%, and between the Chilean BVDV-1j viruses ranged from 98 to 100%. The sequence identity of BVDV-1j viruses, when viruses from other regions of the world were included (KS86ncp, 190CP and Deer-GB1), ranged between 97 and 100%. The sequence identity between the Chilean BVDV-1b viruses with the Brazilian BVDV-1b viruses ranged from 83 to 86%, and the sequence identity between the Chilean BVDV-1b viruses and the Argentinean BVDV-1b viruses ranged from 81 to 86%.

**Fig. 4:**
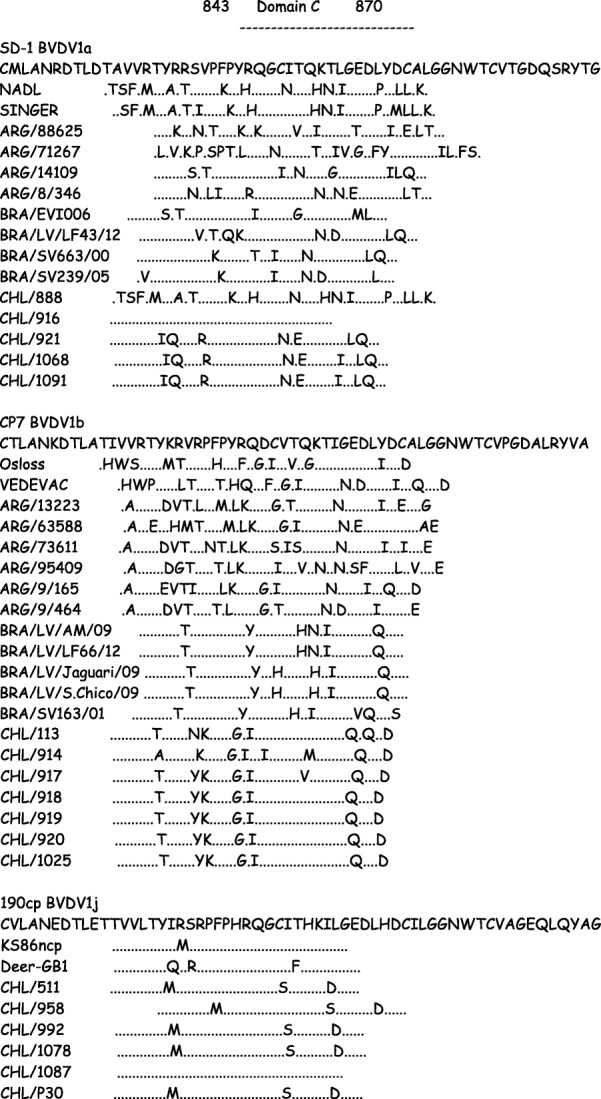
Alignment of partial amino acid sequences of E2 of BVDV-1a, BVDV-1b and BVDV-1j subgenotypes of BVDV. The aligned sequences correspond to amino acids from 832 to 891. Dots represent identical amino acids to the standard sequence of the corresponding subgenotype (BVDV-1a: SD-1, BVDV-1b: CP7, and BVDV-1j: 190cp). Uppercase letters represent an amino acid substitution. Domain C (amino acids 843 to 870) is indicated

To analyze the differences of the E2 glycoprotein of the Chilean isolates at the molecular level, the deduced amino acid sequences were aligned and the substitution patterns in or near Domain C (nDomC), an important antigenic region of BVDV located between amino acid positions 843 and 870 of the NADL polyprotein sequence were analyzed (Fig. 4).

The pattern of amino acid substitutions (PAAS) showed that viruses BVDV/1091, BVDV/921 and BVDV/1068 had the same amino acid sequences in Domain C, and zero or one amino acid substitution in the nDomC of the E2 protein, but these viruses showed three amino acid substitutions in Domain C and seven or eight amino acid substitutions in the nDomC of the E2 protein with respect to the BVDV/916 strain. The PAAS between the Chilean BVDV-1b viruses of this study showed zero or three amino acid substitutions in Domain C, and between zero and five amino acid substitutions in the nDomC of the E2 protein. The PAAS between the Chilean and Brazilian BVDV-1b viruses showed eight or ten amino acid substitutions in the nDomC, and seven to sixteen amino acid substitutions in the nDomC between the Chilean and Argentinean BVDV-1b viruses. The PAAS between the Chilean BVDV-1j viruses of this study showed zero or one amino acid substitutions in Domain C, and between zero and three amino acid substitutions in the nDomC of the E2 protein, and the PAAS between the Chilean BVDV-1j viruses and BVDV-1j viruses from other countries was also very similar, with zero to two amino acid substitutions in Domain C, and between zero and five amino acid substitutions in the nDomC of the E2 protein.

## Discussion

*Bovine Viral Diarrhea Virus* is widely spread in Chilean cattle, with a serological prevalence ranging from 59.7 to 69.2% [22, 23], and although an official control of BVDV is not applied in Chile many farmers are using vaccines in order to reduce economic losses. Nevertheless, an effective vaccination strategy against BVDV requires that vaccines reflect the genetic and antigenic types of viruses that infect Chilean cattle.

A survey of the genetic diversity of 35 field isolates infecting cattle in Chile between 2003 and 2007 was conducted by determining the nucleotide sequences of the 5’-UTR. The results showed that three subgenotypes were circulating in Chilean cattle: BVDV-1a (5/35; 14.3%), BVDV-1b (12/35; 34.3%), and BVDV-1j (18/35; 51.4%), with BVDV-1b and BVDV-1j as the predominant subgenotypes. Surprisingly, BVDV-2 was not detected, even though it had been previously detected in Chile [20]. In the study published by Pizarro-Lucero in 2006 [20], phylogenetic analysis from 14 isolates obtained between 1993 and 2001 showed the presence of viruses belonging to subgenotypes BVDV-1a (1/14; 7.1%), BVDV-1b (6/14; 42.9%), BVDV-1c (currently classified as BVDV-1j viruses) (2/14; 14.3%) and BVDV-2a (5/14; 35.7%).

The results of this study showed that the genetic composition of BVDV viruses infecting cattle in Chile has changed over the years. The BVDV-1b subgenotype has been maintained as a predominant subgenotype in the Chilean cattle, and the subgenotype BVDV-1a is present in low number. Nevertheless, BVDV-1j viruses were isolated more frequently in the samples obtained between 2003 and 2007 (51.4%) in comparison with the samples isolated between 1993 and 2001 (14.3%), and BVDV-2 was not circulating in Chilean cattle, or is present in a very low number that was not detected in this study. Furthermore, considering that all the BVDV-1j viruses analyzed except for African isolate M1515A/90 have lost the restriction site for the Xho I enzyme (data not shown), only 2/33 viruses isolated between 1993 and 2001 in Chile belonged to subgenotype BVDV1j (6.1%).

These differences could be a result of immune selection, because it has been described that BVDV-1j viruses showed significant antigenic differences with BVDV-1a and BVDV-1b viruses, mainly used as vaccine strains [12, 20, 33]. In this study, nine of eighteen BVDV-1j viruses were isolated from cattle showing reproductive problems, but the pathogenicity of the BVDV-1j subgenotype was not clear yet. Otherwise, the high similarity shown between the Chilean and Argentinean BVDV-1j viruses (cluster 1j-SAM) suggests a common ancestor for these viruses. The non-obvious association of any subgenotype with geographical location was expected due to the absence of a national control program that could limit the free movement of the BVDV infected animals around the country.

The E2 phylogenetic analysis showed the same typing obtained with the 5’UTR region. Analyzing the phylogenetic tree of E2 (Fig. 2) and the sequence identity of the viruses of the subgenotypes present in Chile (Fig. 3), three different situations can be observed: a) the Chilean BVDV-1a viruses showed greater genetic diversity between them than the viruses of the other subgenotypes. This may be explained by continuous reintroductions of these viruses to national cattle, probably through commercial vaccines, since the viral strains NADL and SD-1 are frequently used as vaccine strains; b) the Chilean BVDV-1j viruses showed low genetic diversity both among themselves and with the BVDV-1j present in other regions of the world, which is expressed in a relatively compact cluster of these viruses in the phylogenetic tree and high sequence identity between them. This low genetic diversity in the most variable protein of the virus between strains isolated from such diverse regions of the world (Chile, Japan, United Kingdom) could be explained by a relatively recent introduction of this viral subgenotype in cattle, which agrees with its low geographic distribution worldwide; and c) the Chilean BVDV-1b viruses of this study grouped into a single cluster, different even from viruses genetically representatives of the viruses that had been circulating in similar period of time in Argentina and Brazil (2001–2009 and 2006–2010, respectively) [34, 35], countries geographically close to Chile. These results could be explained by the presence of BVDV-1b viruses in the Chilean cattle for a long period of time, which would have allowed a process of local evolution. This process of local evolution could generate important antigenic differences between viruses of the BVDV-1b subgenotype but with different evolutionary histories, as reported by Pecora et al., 2014 [34]. This suggests performing studies on the antigenic diversity of the BVDV-1b viruses present in different regions of the world and evaluating the relevance of generating vaccines with local strains of this viral subgenotype.

The pattern of amino acid substitutions in or near Domain C (nDomC) in the E2 region of Chilean viruses of the different subgenotypes showed that most amino acid changes occurred in the same positions, and usually with the same amino acids in viruses of equal subgenotype (Fig. 4).

The Chilean BVDV-1a viruses BVDV/921, BVDV/1068 and BVDV/1091 showed a very similar amino acid sequence, and larger differences with BVDV/916, that showed an identical amino acid sequence with the SD-1 strain. This is similar to the CHL/888 strain that showed identical amino acid sequence with the NADL strain. These results support those observed with molecular phylogeny and pairwise identity matrix, that some of these viruses can be the result of isolation of vaccine strains. The Chilean BVDV-1b viruses showed a very similar amino acid sequence among them and with the viral strain CP7 (USA), and clearly different with the Brazilian BVDV-1b viruses (obtained between 2001 and 2012) and the Argentinean BVDV-1b viruses (obtained between 2006 and 2010). This high variability in and near Domain C, an important antigenic region of BVDV in E2, the immunodominant protein of BVDV, could cause important changes in the antigenicity of these viruses. The Chilean BVDV-1j viruses showed very similar amino acid sequences between them, and also with BVDV-1j viruses not present in Chile (KS86, 190CP and Deer-GB), but clearly different than the viruses that belong to BVDV-1a and BVDV-1b subgenotypes. These results supports those shown previously, that BVDV-1j viruses have very similar antigenicity between them, but have important antigenic differences with viruses of the BVDV-1a, BVDV-1b and BVDV-2 subgenotypes [12, 20, 33].

The results of this study showed that the genetic profile of the BVDV viruses prevalent in Chile is quite different from the virus diversity reported in South America, Europe and North America [9, 17, 34–42]. The high presence of viruses that belong to subgenotype BVDV-1j in Chile and the increase shown over time makes a significant difference with the genetic profile of the virus reported in other countries of the world, including South America, that lead us to consider viruses of the BVDV-1j subgenotype as a possible emergent group of BVDV viruses in cattle.

Although an official control of BVDV is not yet applied in Chile, vaccines are used in many farms, and the current commercial BVDV vaccines are mainly based on BVDV-1a, BVDV-1b or BVDV-2a strains. Nevertheless, although all BVDVs share epitopes of a common pestiviral antigen, antigenic differences have been demonstrated among BVDV types [43], and this is particularly true between BVDV-1a/BVDV-1b and BVDV-1j strains, which have shown major antigenic differences [12, 20, 33].

Further studies should be done to assess if the predominance of BVDV-1j strains in Chile has been maintained over time, reduces the effectiveness of vaccination campaigns, and the need to include BVDV-1j viruses when designing a vaccine for future use in Chile.

## Conclusions

The results of this study showed that the genetic profile of BVDV in Chilean cattle is composed of the subgenotypes BVDV-1a, BVDV-1b, and BVDV-1j, with BVDV-1b and BVDV-1j as the predominant subgenotypes.

The Chilean BVDV-1b viruses grouped into a single cluster, apart from the viruses present in Argentina and Brazil, countries geographically close to Chile. This process of local evolution may have generated antigenic differences between the Chilean BVDV-1b viruses and the viruses used in vaccines.

The high increase shown over time of BVDV-1j viruses in Chile, and the low genetic diversity in the E2 gene, the most variable protein of the virus, both among Chilean BVDV-1j viruses and with BVDV-1j viruses present in other regions of the world, suggests that BVDV-1j is an emergent group of viruses in cattle in Chile, and since they show important antigenic differences with BVDV-1a and BVDV-1b viruses used in commercial vaccines, their inclusion in the vaccines used in Chile should be evaluated.

## Abbreviations

5’UTR: 5’ Untranslated Region
ATCC: American type culture collection
BVDV: *Bovine Viral Diarrhea Virus*
BVDV-1: *Bovine Viral Diarrhea Virus* species 1
BVDV-1a: *Bovine Viral Diarrhea Virus* subgenotype 1a
BVDV-1b: *Bovine Viral Diarrhea Virus* subgenotype 1b
BVDV-1j: *Bovine Viral Diarrhea Virus* subgenotype 1j
BVDV-2: *Bovine Viral Diarrhea Virus* species 2
DEPC: Diethyl pyrocarbonate
DIF: Direct immunofluorescence
E2: Glycoprotein E2
FITC: Fluorescein isothiocyanate
MDBK: Madin-Darby bovine kidney cell
nDomC: Near Domain C
PAAS: Pattern of amino acid substitutions
PCR: Polymerase Chain Reaction
PI: Persistently infected
PID: Percentage identities
RT-PCR: Reverse transcription polymerase chain reaction
